# Combined Punctual and Diffused Monitoring of Concrete Structures Based on Dielectric Measurements

**DOI:** 10.3390/s21144872

**Published:** 2021-07-16

**Authors:** Andrea Cataldo, Raissa Schiavoni, Antonio Masciullo, Giuseppe Cannazza, Francesco Micelli, Egidio De Benedetto

**Affiliations:** 1 Department of Engineering for Innovation, University of Salento, 73100 Lecce, Italy; andrea.cataldo@unisalento.it (A.C.); raissa.schiavoni@unisalento.it (R.S.); antonio.masciullo@unisalento.it (A.M.); giuseppe.cannazza@unisalento.it (G.C.); francesco.micelli@unisalento.it (F.M.); 2 Department of Information Technology and Electrical Engineering (DIETI), University of Naples Federico II, 80125 Naples, Italy

**Keywords:** microwave reflectometry, era 4.0, frequency domain reflectometry, time domain reflectometry, structural health monitoring, concrete, microwave measurements, dielectric permittivity, water content, construction 4.0

## Abstract

This work presents a microwave reflectometry-based system for monitoring large concrete structures (during the curing process and also while the structure is in use), through the combined use of punctual and diffused sensing elements. In particular, the adoption of punctual probes on a reference concrete specimen allows the development of an innovative and accurate calibration procedure, useful to obtain the value of the water content on a larger structure made of the same material. Additionally, a wire-like diffused sensing element can be permanently embedded in buildings and used to monitor the structure along the entire length of the sensing element. The adopted diffused sensing element can be used not only to detect dielectric variation during the curing process, but also throughout the service life of the structure. The combined use of punctual and diffused sensing elements represents an important innovation from a procedural point of view, able to provide detailed and quantitative information on the health status of the structure both during and after construction.

## 1. Introduction

Construction 4.0 indicates the digital transformation of the construction sector, and it includes the introduction of digital solutions to build faster, cheaper and smarter monitoring solutions for maintenance and prognostics [1,2,3,4,5,6,7,8,12]. In this regard, the application of innovative Information and Communication Technology-based monitoring strategies contributes to this goal. Attention must be paid to monitoring in order to ensure the longevity and efficiency of the built environment. Concrete is one of the most used structural materials in the civil engineering industry thanks to its relatively low material cost, high durability and versatility. These properties are intrinsically related to the concrete curing procedure [9] and also to the monitoring and consequent timely intervention in case of any anomaly or degradation phenomena. The possibility to monitor ex-ante and ex-post any structure is a key factor in the era of Construction 4.0 [10,11,12].

As well known, the hardening phase begins immediately after setting, with a series of physical and chemical interactions between cement and mixing water and this phase is considered completed within the first 28 days, period when an accurate monitoring is essential. A premature removal of formworks may lead to an insufficient strength of the structure and to the presence of cracks, ultimately leading to the collapse of the entire structure [13,14]. Concrete curing occurs through water evaporation and re-arrangement of particles within the mix, resulting in a decrease of the concrete volume and in the development of internal possible strains that can cause the so-called shrinkage strain, responsible for the small cracks appearing after the curing process. The adoption of innovative, accurate and reliable techniques for monitoring the concrete curing of a structure is one of the key pillars of Construction 4.0, as any technological innovation is always based on the principles of ensuring the life safety.

On the other hand, an ex-post monitoring through an embedded low-cost diffused sensing element can be able to promptly detect deterioration of the structures, due to age or environmental loads as floods, for example in the case of dams [15].

Starting from these considerations, in this paper, an innovative, real-time, low cost, with portable instrumentation concrete monitoring technique based on microwave reflectometry is proposed, through the combined use of diffuse sensing elements and punctual probes. The goal of the proposed method is twofold: (1) an ex-ante monitoring of concrete curing with PCPs and a specific calibration procedure; and (2) an ex-post monitoring, carried out through diffuse sensing elements, for the detection of dielectric anomalies as a result of degradation or stress of the structure.

With regard to the ex-ante monitoring, in fact, concrete exhibits good performance throughout the service life of the structure when the concrete curing proceeds correctly and completely. For this reason, an innovative and accurate calibration procedure (to be performed in each specific application case) becomes a key element in the concrete curing process, allowing to assess the water content value of the structure by simply considering that of a concrete specimen. Additionally, there are special cementitious compounds that employ additives that reduce the setting time of concrete, for very fast constructions. In these cases, the water content is a useful parameter to consider when analyzing the quality and setting status of concrete.

On the other hand, the continuous ex-post monitoring through permanent, diffuse sensing elements can give early indications of structural problems. In this way, thanks to the detection of electrical impedance variations or dielectric variations caused by degradation phenomena through the diffuse sensing elements, safety measures can be considered in time, and intervention on the structure can be performed immediately.

The present work is organized as follows. Section 2 addresses the motivations of the work and provides an overview of state-of-the-art solutions for monitoring concrete curing. Section 3 describes the basic theoretical background behind the proposed system and Section 4 describes the used experimental setup. Experimental results, with the use of the adopted methodological procedures as an innovative MATLAB algorithm to extrapolate the water content of a large structure, are reported in Section 5. Finally, the conclusions and the future work are outlined in Section 6. Appendix A details the fitting model used to extrapolate the calibration surface.

## 2. State of the Art and Rationale of the Present Work

The properties of concrete are often examined through stress and compression resistance tests on core samples extracted from the structure. Materials are analyzed through techniques to destroy them, which could also cause damage to the concrete building, which is an unintended option. In recent years, the focus is mainly on non-destructive strength evaluation techniques [16,17], which are born as a response of the need for structural damage detection and prevention. There are different types of non-desctructive tests depending on the fundamental principle on which they are based: thermal, acoustic, electrical, magnetic, optical, radiographic and mechanical. Many of these techniques, however, exhibit significant limitations for practical use.

At the current state of the art, calorimetry represents the standard method to monitor the hydration process of concrete: in fact, the hydration balance of cement is exothermic, and for this reason, hydration can be monitored by measuring the heat released by means of an isothermal calorimeter [18,19,20]. This method has an important limitation: especially during the final stage of hydration, the material emits little heat and the technique is not very sensitive. The material also must be placed in a thermostatic enclosure, so calorimetry can only be applied in the laboratory and not in situ [21]. Methods based on plastic optical fibers [22] exploit the transmission power loss due to the fiber micron-bending, which can be associated to the curing process, have the same problem of reduced sensitivity. Other methods use a combination of the Acoustic Emission (AE) Technique with linear and nonlinear ultrasonic/elastic wave spectroscopy [23], but, in this case, the curing cell used for monitoring the hardening process in young concrete is very complex (including AE sensors, thermocouples and compressional and shear transducers). As additional drawback, the sample must be placed in a climate chamber, thus limiting the in situ analysis. Techniques based on electromagnetic probing are widely used for evaluation of concrete curing [24,25,26], because the electrical and dielectric response of a porous material are strongly dependent on the water content of the sample, but, in most cases, sensors working at a single frequency are used [27,28]: the internal water content of the samples could be measured by tracking the changes in the sensor’s resonant frequency. A single-frequency monitoring may not be completely exhaustive compared to a broadband monitoring that provides more information about the electromagnetic interaction between the test signal and the material under test.

Based on these considerations, this work proposes an innovative strategy based to microwave reflectometry for monitoring concrete curing. In particular, a dual sensor system with diffuse sensing element (d-SE) and punctual coaxial probes (PCPs) was used for the intended purposes. The punctual detection is used to identify a calibration procedure using a reference concrete specimen. This procedure allows to relate the data collected in the frequency range of interest to the value of water content. A dedicated algorithm was developed in order to employ the calibration procedure carried out on the specimen, even on the large structure from which the specimen was extracted. This aspect is particularly important because structures of large dimensions cannot be weighed or subjected to the gravimetric procedure: the adoption of a dedicated algorithm that exploits PCPs on a reference concrete specimen allows to circumvent this limit. As an additional detection capability, a long diffused wire-like low-cost sensing element, permanently embedded in buildings, is used to get feedback along the entire profile of the structure and to detect possible dielectric variations both during the curing process and also throughout the service life. The combined use of punctual and diffused can provide detailed and quantitative information on the state of hydration and health of the structure. Ultimately, a broadband and low cost reflectometric system using both PCPs and d-SEs with strategic methodological procedure was validated.

## 3. Theoretical Background

Microwave reflectometry is an electromagnetic (EM) measurement technique, employed for a number of diagnostics and monitoring applications. While one of the major application fields remains soil moisture content monitoring [29,30,31,32], this technique is also largely used for applications such as leak localization in underground pipes [33,34,35]; the characterization of devices [36]; etc.

In microwave reflectometry, an EM test signal is propagated through a sensing element inserted in the system under test (SUT). As a result of the dielectric interaction with the SUT, the test signal is partially reflected towards the measuring instrument. Through the analysis of the reflected signal and through an application-tailored processing, it is possible to retrieve the desired information on the SUT (e.g., water content; structural defects; liquid level; etc.).

Microwave reflectometry-based measurements can be performed either in time domain (time domain reflectometry—TDR) or in frequency domain (frequency domain reflectometry—FDR). Depending on the specific application, one approach may be more suitable than the other. In this work, a combined method is used: in particular, TDR technique is used with d-SEs and FDR technique is used with PCPs.

In TDR measurements, the EM stimulus is usually a step-like signal that propagates along the SE, through the SUT, and any impedance variation, generally due to changes in dielectric properties, causes the partial reflection of the propagating signal. The reflection coefficient ρ is acquired by the TDR instrument, and its value is displayed as a function of time (or as a function of the traveled apparent distance, $d_{app}$). It is expressed as:

(1) $$\rho =\frac{v_{refl}\left(t\right)}{v_{inc}\left(t\right)}$$

where, $v_{refl}\left(t\right)$ is the amplitude of the reflected signal and $v_{inc}\left(t\right)$ is the amplitude of the incident signal.

The quantity $d_{app}$ represents the distance the EM signal would travel, in the same time interval, if it was propagating in vacuum. In fact, the signal propagation velocity inside the medium depends on the dielectric properties of the material in terms of effective relative dielectric permittivity $\varepsilon_{app}$, which describes the interaction between the electromagnetic signal and the SUT. Knowing the physical length of the SE ($L_{real}$), it is possible to obtain the effective dielectric constant of the material in which the probe is inserted through the estimation of the apparent length ($L_{app}$) evaluated from the reflectogram.

(2) $$\varepsilon_{app}={\left(\frac{L_{app}}{L_{real}}\right)}^{2}$$

FDR is often used for the characterization of the dielectric behavior of materials. FDR measurements are generally carried out by connecting the sensing element to a vector network analyzer (VNA), which is used to measure the reflection scattering parameter, $S_{11}\left(f\right)$. In this case, the excitation signal is a sinusoidal signal whose frequency is swept over the desired range of analysis. Clearly, frequency-domain data can also be obtained starting from TDR measurements and applying the Fourier transform. An advantage of the frequency-domain approach is that, in this domain, it is possible to employ well-established error correction models that, through specific calibration procedures (such as the short-open-load calibration), can reduce the influence of systematic errors [37,38].

## 4. Materials and Methods

### 4.1. Experimental Setup

**Figure 1:**
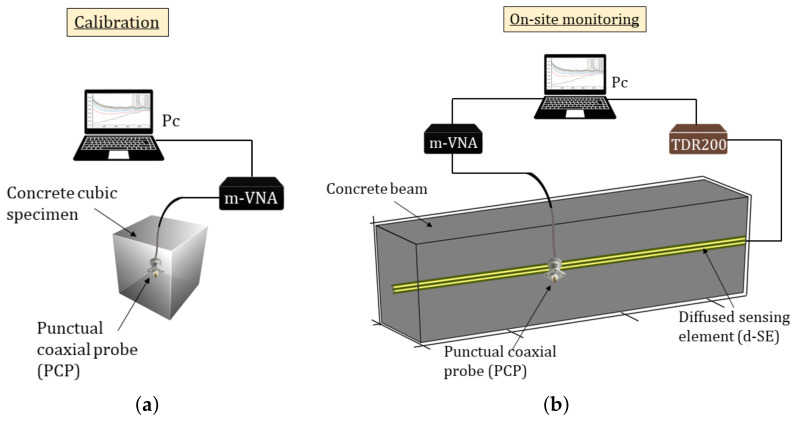
Sketch of the experimental measurement setup: (**a**) reference concrete specimen with an embedded PCP; (**b**) concrete beam with embedded a PCP and a d-SE.

For the validation of the proposed monitoring system, two structures were prepared from the same concrete mix: (1) a beam with dimensions 300 cm × 25 cm × 25 cm; and (2) a cubic specimen with sides 15 cm. Figure 1 shows a sketch of the experimental setup.

As mentioned in the Section 1, two types of sensing element were used to implement the proposed monitoring system: a PCP and a d-SE.

**Figure 2:**
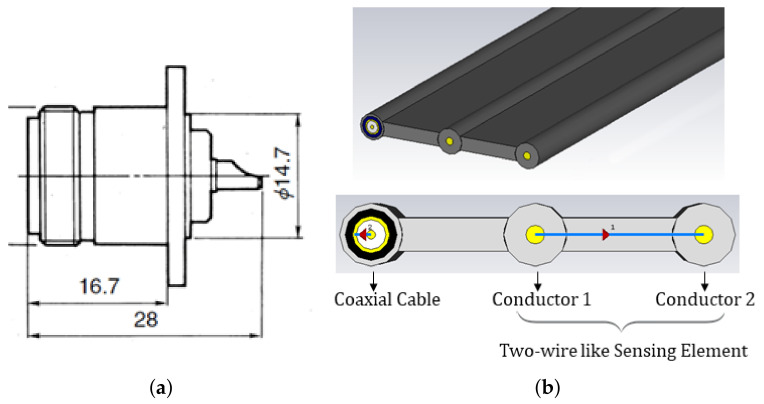
Sketch of the adopted sensing elements: (**a**) Punctual Coaxial Probe (PCP) with dimension in mm; (**b**) Diffused Sensing-Element (d-SE).

Figure 2a shows a sketch of the PCP. The PCPs used were N-type connectors. Clearly, this probe configuration could provide information on a very limited volume. As detailed in the following section, this probe was used for punctual, off-site measurements.

One PCP was used in the specimen and one in the beam. As detailed later, the PCP in the specimen allowed us to obtain a calibration curve from measurements on the specimen, which could later be used for on-site measurements. On the other hand, the PCP in the beam was used to verify that the MR response of the specimen was consistent and in accordance with the specimen results.

Figure 2b shows a sketch of the diffused sensing element. This consisted of two conductors that ran parallel to each other and were mutually insulated through a plastic jacket. This sensing element configuration, d-SE, provided a diffused monitoring of the SUT. In practical applications, the d-SE was permanently embedded in the SUT; one end of the d-SE remained accessible for carrying out measurements.

Generally, d-SEs can be permanently embedded in buildings, structures, infrastructures at the time of construction, and they are capable to return a response based on diffuse dielectric characteristics of the structure, also particularly useful for early identification of destructive phenomena.

From Figure 2b, it can be noticed that the diffused SE also included a coaxial cable, parallel to the two wires. The coaxial cable served the purpose of evaluating the actual length of the SE. In fact, because the dielectric characteristics of the coaxial cable are known and independent of the concrete, by propagating the TDR signal along the coaxial cable, it is possible to evaluate the actual distance of the SE from (2), as reported in [33]. Clearly, on a laboratory scale, this step may be avoided, since the physical length of the SE can be easily measured; however, in practical applications in building structures, the length of the embedded SEs is not necessarily known in advance.

As described later in Section 5.5, however, the coaxial cable could also be sued for ex-post monitoring for detecting possible mechanical stress and consequent deformations of the concrete structures.

**Figure 3:**
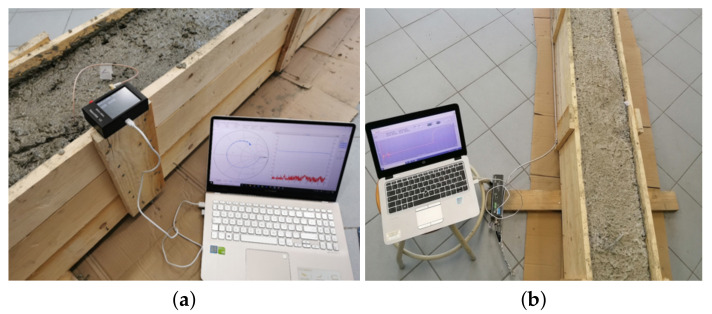
Experimental setup for measurements on the concrete beam: (**a**) nanoVNA with PCP for FDR measurements; (**b**) TDR with d-SE for TDR measurements.

Figure 3 shows the pictures of the experimental setup. Two measuring instruments were used: a Campbell-Scientific TDR200 (Leicestershire, UK) (operating in the time domain) and a miniaturized VNA operating in the frequency domain (nanoVNA) (Amsterdam, The Netherlands).

The nanoVNA is a compact, low-cost VNA (dimensions 15 cm × 10 cm × 6 cm), with 50 kHz–3 GHz frequency range. One of the advantages of FD measurements is the possibility of carrying out a preliminary calibration procedure through measurements on standard electrical loads (short, open, load-SOL), thus allowing to reduce the influence of systematic errors. To this purpose, a preliminary calibration procedure was carried out on each of the PCPs, before inserting them in the concrete samples. The calibration data were stored in the software of the miniaturized VNA thus compensating for each daily measurement.

The TDR200 is a low-cost, portable TDR measuring instrument, with approximate dimensions 22 cm × 5 cm × 11 cm. The TDR200 generates a step-like voltage signal, with a 200-ps rise time, which corresponds to a frequency bandwidth of approximately 1.7 GHz.

It is important to mention that the presence of steel in the concrete beam did not affect the investigated method and the measurement results; this was also verified through specific full-wave simulations carried out through CST Microwave Studio software (not reported here for the sake of brevity).

### 4.2. Methodological Procedures and Description of the Experiments

**Figure 4:**
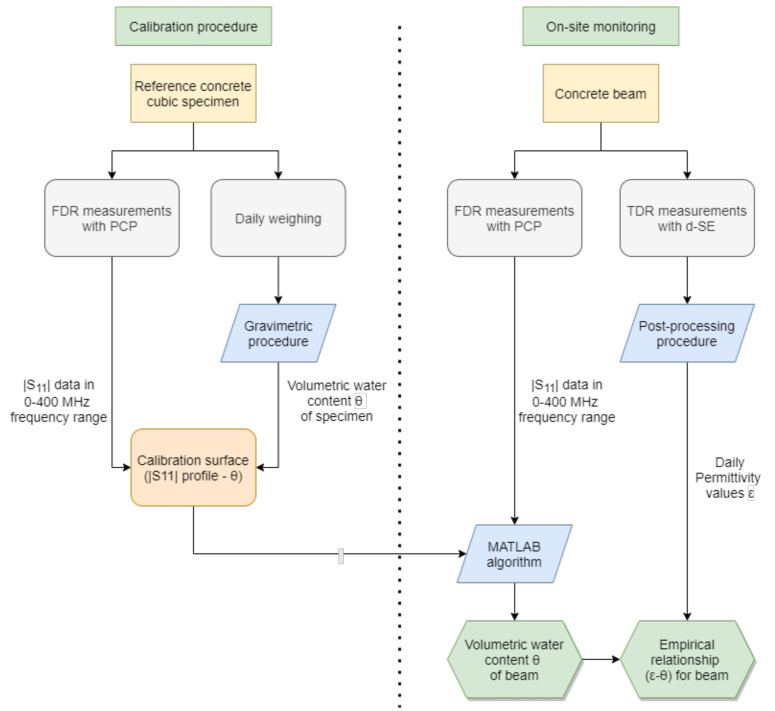
Diagram of the combined monitoring procedure: Preliminary Calibration Procedure (**left**) and procedure for on-site monitoring procedure (**right**).

The overall experimental procedure is outlined in Figure 4. It consisted of two major phases: a calibration procedure (a) and the on-site monitoring procedure (b).

The first step required us to measure the $|S_{11}\left(f\right)|$ through FDR measurements on the PCP inserted in the cubic specimen. These measurements were carried out daily over a 28-day period (in fact, generally, after this period, more than 90% of the overall mechanical strength of a structure had developed). During the same observation period, the specimen was weighed on a daily basis, and the corresponding volumetric water content (θ) was evaluated through the gravimetric method. After 28 days, the obtained data were used to obtained an empirical calibration surface relating the θ values of the specimen to the $|S_{11}\left(f\right)|$ measurements.

The second phase of the procedure outlined in Figure 4 relates to the on-site monitoring. The calibration surface obtained in the first step was used to infer the water content of the beam, starting from on-site FDR measurements of the reflection scattering parameter obtained from the PCP in the beam, $|S_{11,meas}\left(f\right)|$. As detailed in the following section, the evaluation of θ was carried out through a specifically-developed Matlab algorithm. The algorithm extrapolated from the calibration surface the $|S_{11}\left(f\right)|$ curve that best fit $|S_{11,meas}\left(f\right)|$, and returned the unknown θ value.

Finally, the θ values of the beam could also be associated to the distributed dielectric characteristics of the beam, through TDR measurements on the d-SE. This allowed us to obtain a diffused monitoring of the beam, starting from the combination of punctual and elongated sensing elements.

## 5. Experimental Results

### 5.1. Preliminary Calibration Procedure

**Figure 5:**
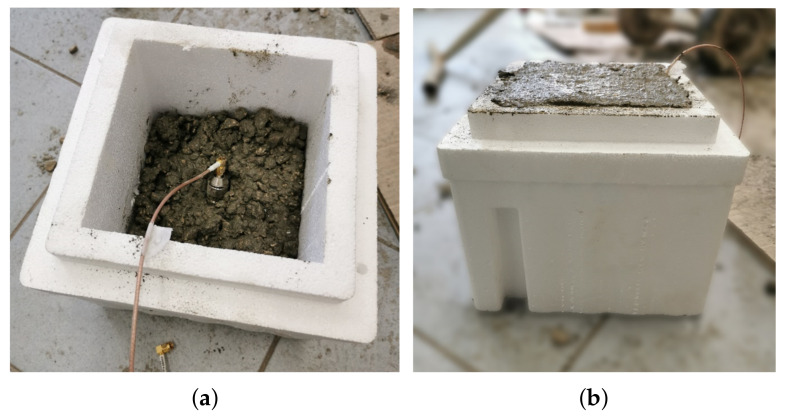
Picture of the preparation of the reference concrete specimen. The PCP is inserted in the fresh mixture.

Figure 5 shows a picture of the preparation of the cubic specimen with an embedded PCP. The reference specimen was used to carry out the calibration procedure through which the measurements made on the beam was correlated to the water content of the beam structure.

The θ value of the cubic specimen during the 28-days period, was assessed through the gravimetric method. The total weight varied from 7747.6 g (in the first day) to 7536.5 g on the 29th day. The last weighing, after drying the specimen in an oven, was equal to 7325 g. This condition was considered as a reference dried condition (i.e., θ = 0 %) for the sample. The measured θ values are summarized in Table 1.

**Figure 6:**
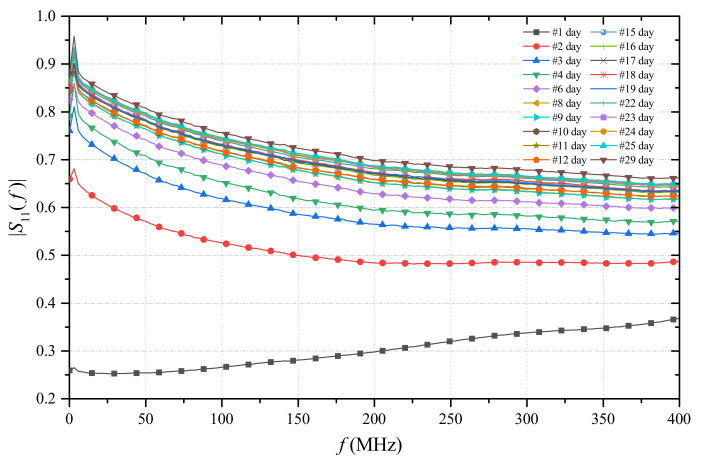
Trend of the $S_{11}$ with each passing day in the cubic specimen case in 0–400 MHz.

During the observation period, the $|S_{11}\left(f\right)|$ of the PCP inserted in the specimen was measured through the nanoVNA. Figure 6 shows the obtained results. It can be noticed that, as the hydration proceeded, the $|S_{11}|$ curves shifted towards higher values (and, accordingly, the water content decreased). The measurements were acquired in the 0–400 MHz frequency range, since in this frequency range, a better sensitivity was observed. For FDR measurements, the specifications of the nanoVNA provided an instrumental uncertainty of 2%.

Each $|S_{11}\left(f\right)|$ curve was associated to the corresponding θ value of the specimen. This resulted in a three-dimensional graph. The resulting 3D scattered plot was fitted with MATLAB Curve Fitting Toolbox (see Appendix A), in order to obtain a calibration surface:

(3) $$\Theta =\Theta (f,|S_{11}\left(f\right)|)$$

**Figure 7:**
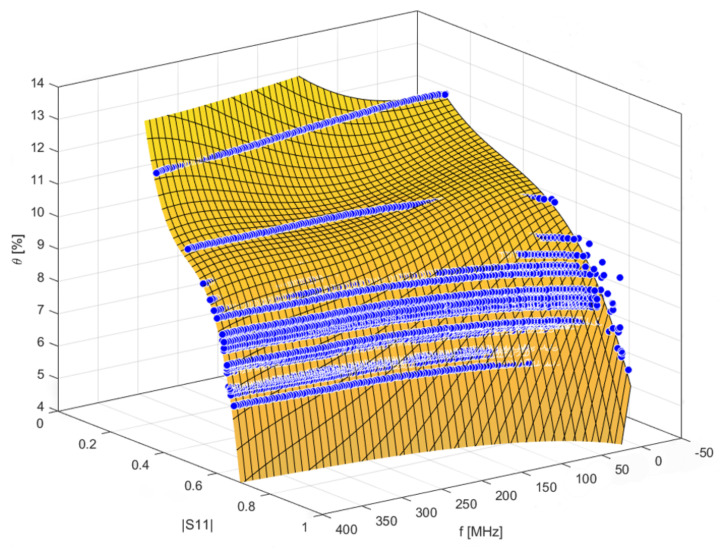
Graphical representation of the calibration surface.

The graphical representation of the fitting is shown in Figure 7.

### 5.2. Comparison of Results Obtained from the Specimen and from the Beam

**Figure 8:**
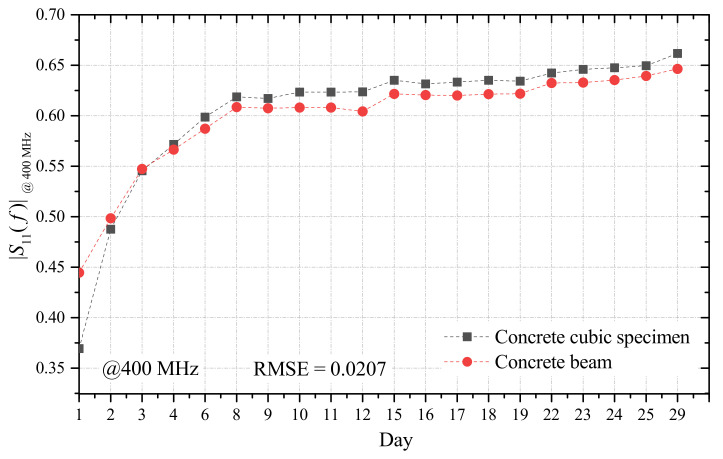
Comparison of the $|S_{11}|$, measured at 400 MHz, during the observation period: from the PCP in the specimen (black squares) and from the PCP in the beam (red circles).

To demonstrate that the calibration surface obtained from the specimen could also be used for the beam monitoring, it was important to verify that the frequency behavior found on the reference cubic specimen was consistent with that of the beam. To this purpose, during the 28-day observation period, FD measurements were carried out also through the PCP inserted in the concrete beam, as shown in Figure 3a. Overall, during the observation period, a similar trend of the $|S_{11}|$ was observed from the PCP in the specimen and from the PCP in the beam. For the sake of example, Figure 8 shows the comparison of the $|S_{11}|$, measured at 400 MHz, during the observation period: specimen (black squares) and beam (red circles). To assess the agreement between the two curves, the root mean square error (RMSE) was used a figure of merit. The obtained value was 0.0207. This low value indicates that the curves obtained on the specimen were representative of those on the beam and that it was safe to assume that the two structures exhibited the same dielectric characteristics.

### 5.3. Procedure for On-Site monitoring

**Figure 9:**
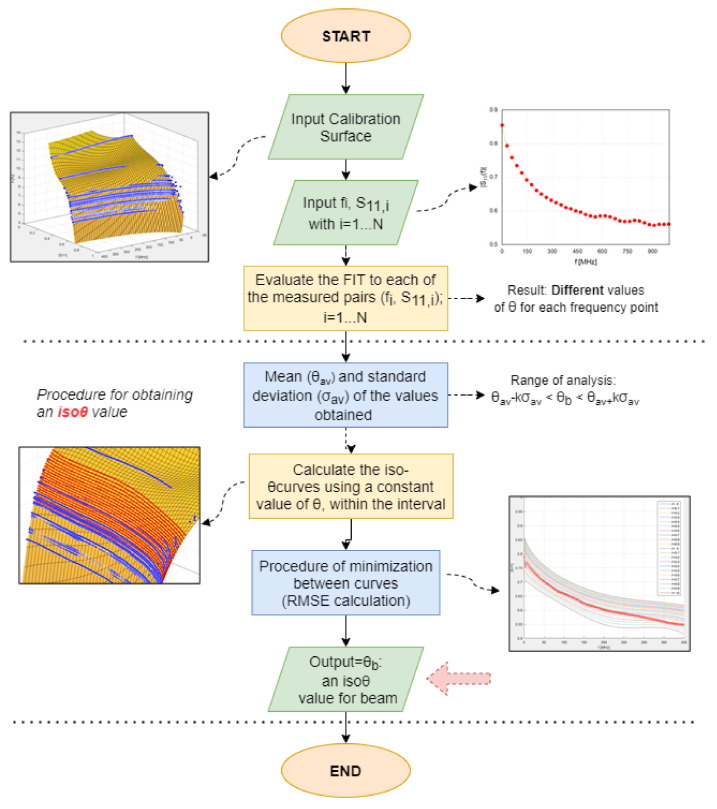
Flowchart of MATLAB algorithm developed to extrapolate the volumetric water content of the beam using the calibration surface.

As mentioned in Section 4.2, a dedicated automated algorithm was developed to be used on site, for evaluating the volumetric water content of the beam, $\theta_{b}$, using the calibration surface. Figure 9 summarizes the major steps of the procedure; in this section, the procedure is applied to the considered experimental case.

Let us suppose that we wanted to assess $\theta_{b}$ at a generic day. The first step consisted in measuring the $|S_{11}\left(f\right)|$ from the PCP in the beam, as shown in Figure 3a. The obtained measurement points were considered as pairs $\left(f_{i},S_{11,i}\right)$ with i=1,…,N, where *N* is the number of frequency points. Through a Matlab routine, we singled out from the calibration surface the moisture content values, $\theta_{i}$, that corresponded to each of the measured $\left(f_{i},S_{11,i}\right)$ pairs:

(4) $$\theta_{i}=\Theta \left(f_{i},S_{11,i}\right)\;\;\;i=1,\ldots ,N$$

Then, the algorithm calculated the average of the moisture content values obtained from the previous step:

(5) $$\theta_{av}=\frac{1}{N}\sum_{i}^{N}\theta_{i}$$

Then, from the calibration surface (Θ), we calculated the iso-θ curves that corresponded to a constant value of θ, with a step of 0.1%, in an interval around the value of $\theta_{av}$.

The final step consisted in comparing the measured $|S_{11}|$ curve with each of the iso-θ. In fact, the measured $|S_{11}|$ curve could be considered as an iso-θ curve for which the θ value was unknown. The aforementioned comparison was carried out in terms of RMSE. In particular, the algorithm automatically identified the iso-θ curve that provided the lowest RMSE with respect to the measured $|S_{11}|$. The θ value that minimized the RMSE was the volumetric water content associated to the beam, $\theta_{b}$.

**Figure 10:**
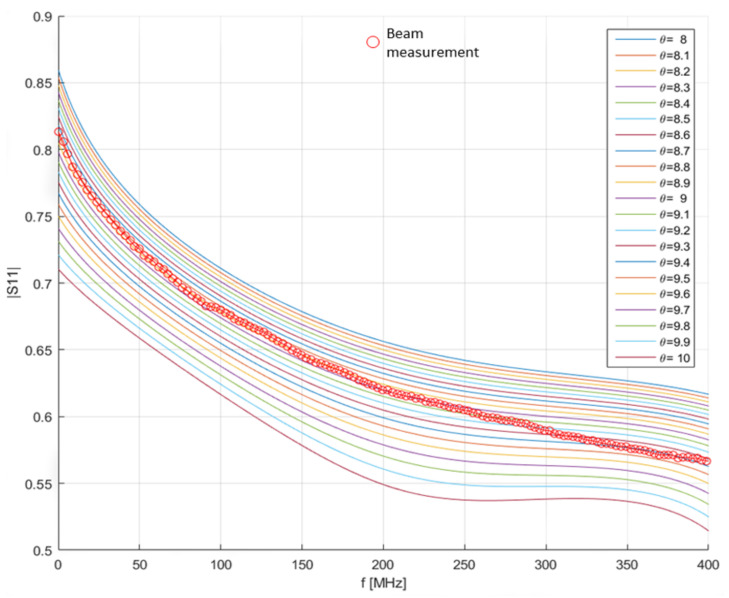
Comparison of the measured $|S_{11}\left(f\right)|$ (curve with red circles) and the iso-θ curves obtained from Θ surface.

Figure 10 shows the iso-θ curves obtained in the considered experimental case. The curve with circles indicates the measured $|S_{11}|$. The lowest RMSE between the measured $|S_{11}|$ and the iso-θ curves corresponded to a volumetric water content $\theta_{b}$ of 9%. This highlighted the great potential of this method. In fact, any structure (even a large one) could be monitored in terms of water content using a reference specimen made from the same concrete mix and two (or more) PCPs.

### 5.4. TDR Measurements for On-Site Monitoring

The proposed monitoring systems also included TDR measurements with a diffused sensing element (d-SE), as reported in Figure 2 and Figure 3. This setup is useful for obtaining, with a single d-SE, distributed measurements on the beam.

**Figure 11:**
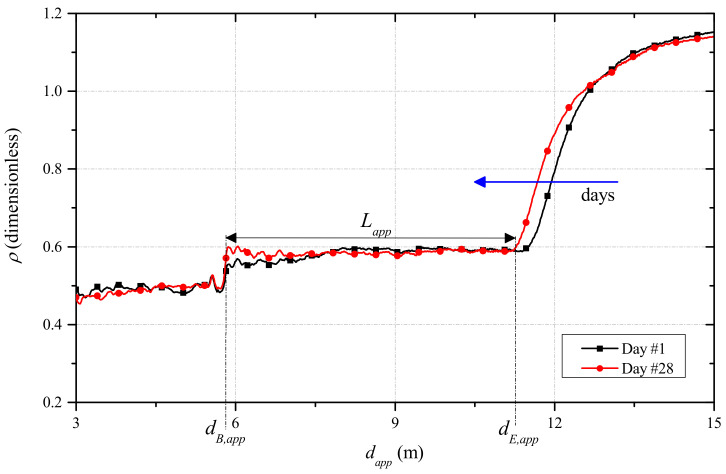
TDR reflectograms acquired through the d-SE, for the concrete beam on day #1 and #28.

TDR measurements were carried daily throughout the observation period. Figure 11 shows the TDR reflectograms acquired from the d-SE, on the first and on the last days of the observation period. From the reflectogram, it was possible to identify the beginning and the end of the SE (denoted respectively by $d_{B,app}$ and $d_{E,app}$, while the portion of the graph before and after these two values referred to portion outside the beam). The corresponding apparent length of the SE, $L_{app}$, could be calculated as

(6) $$L_{app}=d_{E,app}-d_{B,app}$$

It should be mentioned that the abscissae $d_{B,app}$ and $d_{E,app}$ could be identified automatically through the so-called derivative method (not discussed herein for the sake of brevity): in fact, the first derivative of the reflectogram emphasizes the variation that correspond to these points and facilitates the evaluation [33]. As can be seen from Figure 11, because of the hydration process, $d_{E,app}$ shifted towards lower values. This indicates that the apparent length $L_{app}$ of the d-SE decreased with decreasing dielectric permittivity $\varepsilon_{app}$ of the SUT. This phenomenon was related to the evaporation of water as the hydration proceeded.

**Figure 12:**
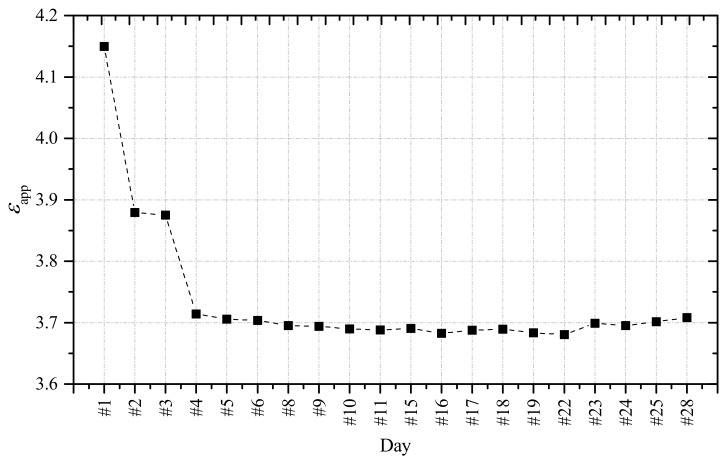
Trend of $\varepsilon_{app}$ during the hydration process.

From each daily TDR reflectogram, the apparent length of the d-SE, $L_{app}$, was evaluated using (6). Employing Equation (2), the corresponding $\varepsilon_{app}$ can be calculated. The variation of $\varepsilon_{app}$ in time is shown in Figure 12. It appears that the value of $\varepsilon_{app}$ varied more quickly in the first 4 days after setting, and then settles after approximately 8–9 days. The reason is that the major physical interaction of water with the concrete mixture occurred during this first period; as a result, the dielectric permittivity of the mixture also underwent the most significant variations during this time interval, before settling to an approximately constant value. This behavior was most probably related also to the variation of mechanical strength developed by the structure, which increased rapidly in the first period after setting the concrete, and then started to settle in time.

**Figure 13:**
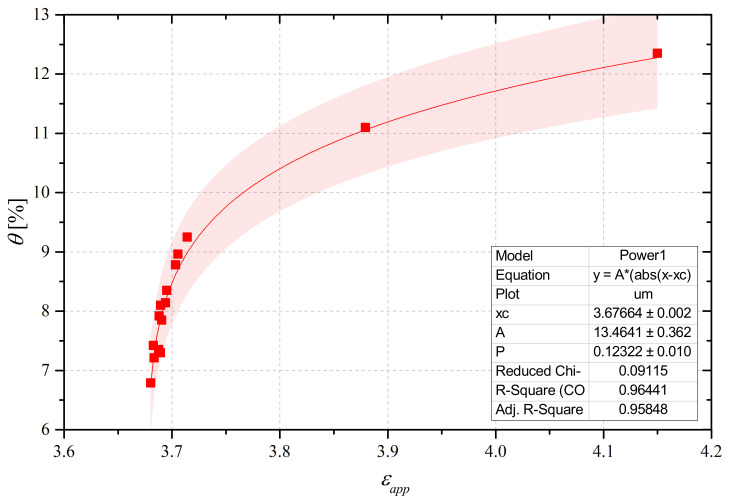
$\varepsilon_{app}$-θ empirical relationship.

The final step, as shown in the flowchart in Figure 4, was to relate the obtained permittivity values to the water content obtained from the algorithm, in order to obtain a new $\varepsilon_{app}$-θ empirical relationship to monitor the structure along a diffuse profile, as shown in Figure 13. This step could be carried out because, as can be seen from Figure 11, the reflectometric signal could be considered practically constant along the length of the d-SE and this meant that there were no significant variations of water content along the beam; hence, we could consider a homogeneous value of θ.

It should be mentioned that in Figure 13, each measurement point was obtained from measurements that were carried out on a daily basis. Because the variations of water content and (consequently) of $\varepsilon_{app}$ are more rapid in the first days after setting, then the $\varepsilon_{app}$-θ points appear sparser in the upper-right portion of the graph.

### 5.5. Mechanical Tests on the Beam

The d-SEs are also particularly useful in the identification of degradation phenomena occurred ex-post along the structure, thanks to their ability to immediately identify a dielectric variation in correspondence of an incipient failure. In this case, alert mechanisms could also be used to promptly intervene in case of incipient cracks.

**Figure 14:**
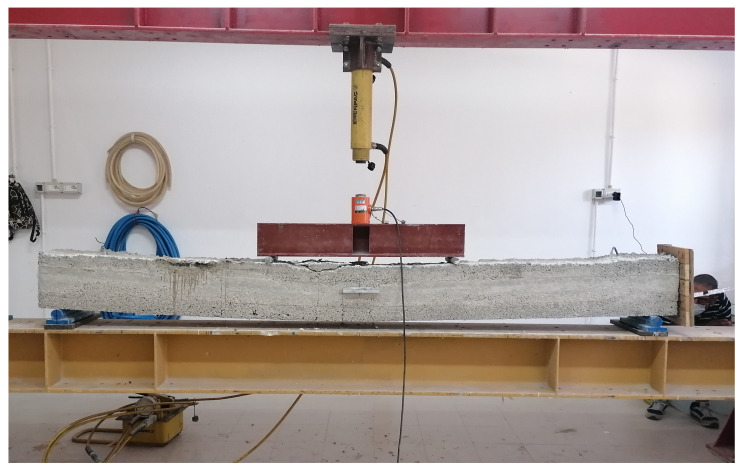
Experimental setup post-mechanical tests on the beam.

Mechanical tests on the beam were carried out for validation purposes of ex-post concrete strength monitoring. The tests were conducted to analyze whether the diffuse sensor cable is sensitive to mechanical deformation. The beam was subjected to several tests with concentrated weights varying from 1000 kgf to 9000 kgf, with an increase of 1000 kgf at each test. More specifically, the beam was tested under four-point bending with a net span of 2.8 m and a shear span of 0.95 m. Load was applied by means of a 100-ton hydraulic jack connected to a manual pump and recorded with a 30-ton load cell. Figure 14 shows a picture of the test setup.

**Figure 15:**
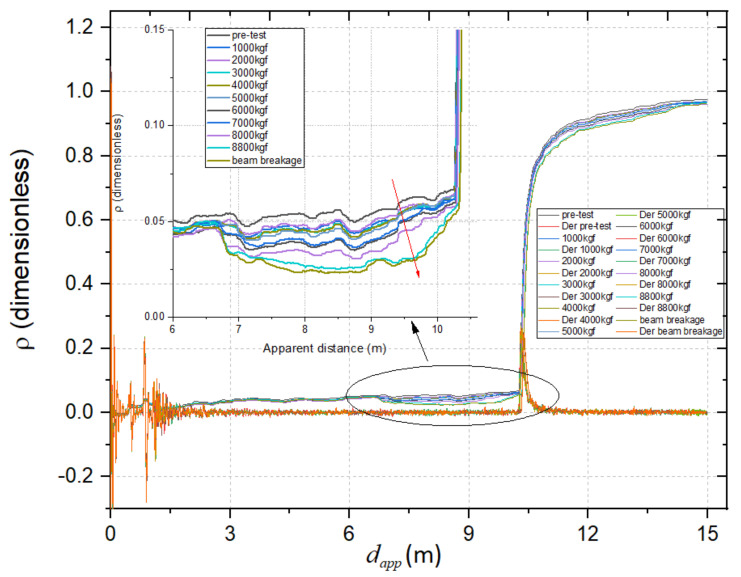
TDR reflectograms and first derivatives acquired through the coaxial cable of the d-SE, for the concrete beam in the different compression conditions.

During the mechanical test, also TDR measurements were carried out on the d-SE. As explained in Section 4.1, the d-SE also includes a coaxial cable, useful for evaluating the actual length of the SE but also sensitive to deformation and/or compression phenomena. As known from the theory, the impedance of the coaxial cable depends on the diameter of the inner and outer conductors, for which the deformation of the cable causes a variation of electrical impedance that we immediately identify from the measurements. As shown in Figure 15, as the weight applied to the beam increased, the reflection coefficient decreaseds, and the apparent distance increased, indicating the deformation and bending of the cable.

These mechanical tests highlighted how the diffuse sensing element could detect any degradation phenomena in time, for accurate ex-post monitoring of the strength and health of the concrete.

## 6. Conclusions and Future Works

In this work, a combined punctual and diffused approach for smart monitoring of concrete structures was presented. The use of punctual sensors on a reference concrete specimen allowed to implement an innovative procedure for extrapolating the water content of large structures, based on measurements carried out on small specimens. This is particularly useful for allowing accurate monitoring of the hydration process of concrete structures. While pouring the concrete mix for a building, in fact, it is easy to use the same concrete mix to obtain a small sample on which to perform the needed measurements and the gravimetric procedure (not feasible on large concrete structures). Hence, a dedicated algorithm was developed to relate on-site measurements to a water content value using the calibration surface obtained from the specimen having the same dielectric properties as the structure to be monitored.

Finally, the simultaneous use of d-ES also allows to monitor ex-post the large concrete structure throughout its service life, both in terms of water content and possible mechanical deformation.

Future work will be dedicated to the comparison with other traditional measurement techniques and to the possibility of relating the water content data to the strength properties developed by the structure during the hydration period.

## Figures and Tables

**Table 1:** Volumetric water content percentage in the concrete specimen throughout the observation period.

Day	θ [%]	Day	θ [%]	Day	θ [%]	Day	θ [%]
1	12.52	2	10.55	3	9.67	4	9.25
6	8.78	8	8.35	9	8.14	10	8.10
11	7.92	12	7.85	15	7.45	16	7.42
17	7.35	18	7.30	19	7.21	22	6.79
23	6.75	24	6.69	25	6.57	29	6.27

**Figure A1:**
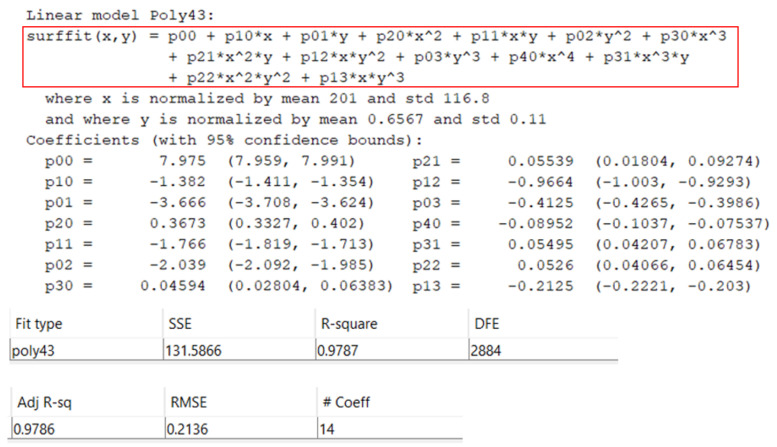
Description of the fit function and of the resulting coefficients.

## Data Availability

Not applicable.

## Appendix A. Fitting

To fit all points, a 4th-degree polynomial and a 3rd-degree polynomial were chosen, for the *f* axis and for the $|S_{11}|$ axis, respectively.
